# Interleukin-1 Stimulates β-Cell Necrosis and Release of the Immunological Adjuvant HMGB1

**DOI:** 10.1371/journal.pmed.0030017

**Published:** 2005-12-20

**Authors:** Sarah A Steer, Anna L Scarim, Kari T Chambers, John A Corbett

**Affiliations:** 1The Edward A. Doisy Department of Biochemistry and Molecular Biology, St. Louis University School of Medicine, St. Louis, Missouri, United States of America; Harvard Medical SchoolUnited States of America.

## Abstract

**Background:**

There are at least two phases of β-cell death during the development of autoimmune diabetes: an initiation event that results in the release of β-cell-specific antigens, and a second, antigen-driven event in which β-cell death is mediated by the actions of T lymphocytes. In this report, the mechanisms by which the macrophage-derived cytokine interleukin (IL)-1 induces β-cell death are examined. IL-1, known to inhibit glucose-induced insulin secretion by stimulating inducible nitric oxide synthase expression and increased production of nitric oxide by β-cells, also induces β-cell death.

**Methods and Findings:**

To ascertain the mechanisms of cell death, the effects of IL-1 and known activators of apoptosis on β-cell viability were examined. While IL-1 stimulates β-cell DNA damage, this cytokine fails to activate caspase-3 or to induce phosphatidylserine (PS) externalization; however, apoptosis inducers activate caspase-3 and the externalization of PS on β-cells. In contrast, IL-1 stimulates the release of the immunological adjuvant high mobility group box 1 protein (HMGB1; a biochemical maker of necrosis) in a nitric oxide-dependent manner, while apoptosis inducers fail to stimulate HMGB1 release. The release of HMGB1 by β-cells treated with IL-1 is not sensitive to caspase-3 inhibition, while inhibition of this caspase attenuates β-cell death in response to known inducers of apoptosis.

**Patient Summary**

**Background**

Type 1 diabetes (also called autoimmune diabetes or juvenile diabetes) is an autoimmune disease. For unknown reasons, at some point in childhood or adolescence, the body's own immune system starts attacking and destroying the insulin-producing islet cells in the pancreas. Once the majority of islet cells are destroyed, patients can no longer produce insulin to regulate their blood sugar and must depend on strict diets and insulin injections. Scientists are trying to understand the early events during the development of the disease. There are two fundamentally different kinds of cell death in cells of higher animals and humans, called apoptosis and necrosis. Apoptosis (also called programmed cell death) is an organized, clean way in which cells die without spilling their contents and without causing an inflammatory immune response. They are simply gobbled up by other cells that serve as the body's garbage collectors. Necrosis, on the other hand, is a more messy process and one that does activate the immune system and cause local inflammation.

**Why Was This Study Done?**

The scientists who did this study are interested in the early stages of islet cell death. Specifically, they wanted to know whether islet cells during the early events of autoimmune diabetes die via apoptosis or necrosis. Earlier experiments to address this question had yielded no clear-cut results.

**What Did the Researchers Do and Find?**

All the experiments for this study were done in cultured cells in the laboratory. For the most part, the researchers used rodent islet cells, and then they confirmed the crucial finding in human islet cells. They grew the cells under conditions that resembled, to the best of their knowledge, the early stages of diabetes, which caused some of the cells to die. They then did a variety of tests to see whether that cell death was through apoptosis or necrosis, and the results showed that the latter was the case. They also identified some of the key factors involved in promoting and executing the necrosis process.

**What Does This Mean?**

One must always be careful to extrapolate from laboratory results like these. With this caveat, the results suggest that early in the development of diabetes cells die by necrosis, and they point to some of the key factors involved. These are important results that will inform future studies toward the goal of understanding autoimmune diabetes well enough to prevent or stop its development.

**Where Can I Find More Information Online?**

The following Web sites provide information on autoimmune diabetes.

MedlinePlus pages on type 1 diabetes:

http://www.nlm.nih.gov/medlineplus/ency/article/000305.htm

Web site of the Juvenile Diabetes Research Foundation:

http://www.jdrf.org/index.cfm?page_id=101982

Pages on type 1 diabetes from the Canadian Diabetes Association:

http://www.diabetes.ca/Section_About/type1.asp

Type 1 diabetes pages from the UK National Institute for Health and Clinical Excellence:

http://www.nice.org.uk/page.aspx?o=213575

UK National Diabetes Information Clearinghouse:

http://diabetes.niddk.nih.gov/index.htm

American Diabetes Association Web site:

http://www.diabetes.org

**Conclusions:**

These findings indicate that IL-1 induces β-cell necrosis and support the hypothesis that macrophage-derived cytokines may participate in the initial stages of diabetes development by inducing β-cell death by a mechanism that promotes antigen release (necrosis) and islet inflammation (HMGB1 release).

## Abstract 2

Results from rodent and human cells suggest that necrosis of β-cells plays a role in the early stages of type 1 diabetes.

## Introduction

Insulin-dependent diabetes is an autoimmune disease characterized by selective destruction of insulin-producing β-cells found in pancreatic islets of Langerhans [1]. The development of diabetes is characterized initially by insulitis, in which leukocytes migrate to and invade islets. This is followed by overt diabetes, distinguished by insulin insufficiency due to the destruction of a majority of β-cells [2,3]. Cytokines produced by inflammatory leukocytes and resident and inflammatory macrophages are believed to contribute to the loss of β-cell function and viability during the development of autoimmune diabetes [4,5]. Interleukin (IL)-1 was identified as the active component of conditioned medium derived from activated human mononuclear cells that causes an inhibition of insulin secretion and the loss of islet cell viability, provided the initial evidence supporting a role for cytokines in the pathogenesis of autoimmune diabetes [6,7].

The mechanisms by which cytokines impair β-cell function have been described in detail. Alone or in combination with interferon (IFN)-γ and tumor necrosis factor (TNF), IL-1 has been shown to stimulate β-cell expression of inducible nitric oxide synthase (iNOS), and the resulting production of nitric oxide impairs β-cell oxidation of glucose to CO_2_, reduces the activity of the Krebs cycle enzyme aconitase, and reduces islet ATP levels by more than 4-fold [8,9]. Importantly, glucose-induced insulin secretion is dependent on β-cell depolarization and Ca^2+^ entry, events that are mediated by the inhibition of ATP-sensitive K^+^ channels due to the accumulation of ATP that is produced by mitochondrial oxidation of glucose to CO_2_ [10,11]. The targeted disruption of mitochondrial oxidation resulting in the reduction of cellular levels of ATP is one mechanism by which nitric oxide impairs glucose-stimulated insulin secretion. The damaging actions of cytokines on β-cell function appear to be selective for β-cells, as IL-1 fails to impair the ability of α-cells to oxidize glucose to CO_2_ [12]. Furthermore, β-cells are the primary islet-cell source of iNOS in response to cytokine treatment [13,14]. The macrophage-derived cytokine IL-1 is the major regulator of iNOS expression by β-cells, IL-1 alone is sufficient to stimulate iNOS expression by rat β-cells, and combinations of IL-1 and IFN-γ stimulate β-cell expression of iNOS in most mouse strains and in human islets [4,9,15]. Biochemical and genetic evidence supports a primary role for nitric oxide as the major mediator of cytokine-induced β-cell damage. The NOS inhibitors N^G^-monomethyl-L-arginine (NMMA) and aminoguanidine (AG) prevent the inhibitory actions of cytokines on insulin secretion, glucose oxidation, and aconitase activity by rat, mouse, and human islets [4,8,9]. In addition, islets isolated from iNOS-deficient mice are resistant to the inhibitory actions of cytokines on insulin secretion [16], and expression of iNOS under control of the rat insulin promoter results in the development of diabetes in a nitric oxide-dependent manner that occurs in the absence of insulitis [17].

While autoimmune diabetes is characterized by selective destruction of β-cells, the biochemical mechanisms by which β-cells are killed are poorly defined. In vitro studies using isolated islets and primary β-cells have shown that nitric oxide is a primary mediator of impaired β-cell function in response to cytokine treatment; however, the role of this free radical as a mediator of β-cell death and the mechanism of cell death has been debated. Much of this debate has centered on whether cytokine-induced islet cell death is apoptotic or necrotic and if nitric oxide is a mediator [18–20]. Early studies by Kolb and coworkers demonstrated that nitric oxide is responsible for IL-1-stimulated DNA damage in β-cells [21]. More recently, cytokines have been reported to induce β-cell apoptosis by a process that requires prolonged exposures of 7–9 d and that appears to occur by nitric oxide-independent mechanisms [19,22]. Similar to the inhibitory actions of cytokines on β-cell function, the macrophage-derived cytokine IL-1 appears to be the primary regulator of cytokine-induced β-cell death, be it by necrosis or apoptosis [18,19,23].

In classical terms, apoptosis is defined as programmed cell death by a pathway that removes unwanted cells in the absence of an inflammatory response. It is a highly organized, energy-dependent process that is characterized by caspase activation, the ordered cellular degradation of proteins and organelles, and maintenance of plasma membrane integrity. The final result is noninflammatory phagocytosis of the dying cell, which minimizes leakage of cellular contents and inflammation [24,25]. In contrast, necrosis is an inflammatory process that is characterized by the loss of membrane integrity and the leakage of cellular contents into the extracellular space. It is an unregulated form of cell death that does not require energy or caspase activation [26]. When considering the role of macrophage-derived cytokines such as IL-1 in the context of the initiation of autoimmune diabetes, one would anticipate that this cytokine would kill β-cells by a mechanism that would promote inflammation. In this report, we compare the effects of IL-1 with known inducers of apoptosis on β-cell death.

## Methods

### Materials and Animals

Sprague Dawley rats were purchased from Harlan (Indianapolis, Indiana, United States). RINm5F (rat insulinoma) cells were obtained from Washington University Tissue Culture Support Center (St. Louis, Missouri, United States). RPMI 1640 containing L-glutamine, CMRL-1066 tissue culture medium, L-glutamine, penicillin, and streptomycin were obtained from GIBCO-BRL (Grand Island, New York, United States). FCS was obtained from Hyclone Laboratories (Logan, Utah, United States). Human recombinant IL-1β was obtained from Cistron Biotechnology (Pine Brook, New Jersey, United States). Sodium (Z)-1(*N,N*-diethylamino) diazen-1-ium-1,2-diolate (DEANO) was obtained from Alexis (San Diego, California, United States). Enhanced chemiluminescence reagent was purchased from Amersham Pharmacia Biotech (Piscataway, New Jersey, United States). MTT (3-[4,5-dimethylthiazol-2-yl]-2,5-diphenyl tetrazolium bromide), neutral red, DAPI (4′,6-diamidino-2-phenylindole dihydrochloride), camptothecin, and staurosporine were obtained from Sigma (St. Louis, Missouri, United States). In Situ Cell Death Detection kit was obtained from Roche (Indianapolis, Indiana, United States). Caspase-3 Fluorometric Assay was obtained from R&D Systems (Minneapolis, Minnesota, United States). Annexin V-FITC Apoptosis Detection kit and the caspase-3 inhibitor Z-DQMD-CHO were obtained from Calbiochem (San Diego, California, United States). Guinea pig anti-human insulin antibody was obtained from Linco Research (St. Louis, Missouri, United States). CY3-conjugated donkey anti-guinea pig antibody and HRP-conjugated donkey anti-rabbit and donkey anti-mouse antisera were obtained from Jackson Immunoresearch Laboratories (West Grove, Pennsylvania, United States). Rabbit anti-mouse HMGB1 antibody was obtained from BD Biosciences Pharmingen (San Diego, California, United States). All other reagents were from commercially available sources.

### Islet Isolation and Cell Culture

Islets were isolated from 250- to 300-g male Sprague Dawley rats by collagenase digestion as previously described [27]. Islets were cultured overnight in complete CMRL-1066 (containing 2 mM L-glutamine, 10% heat-inactivated fetal calf serum, 100 U/ml penicillin, 100 μg/ml streptomycin, and 5.5 mM glucose) at 37 °C under an atmosphere of 95% air and 5% CO_2_ prior to experimentation. For annexin V and terminal nick-end labeling (TUNEL) assays of cell death, rat islets were treated with IL-1 for the indicated times followed by dispersion into individual cells by trypsin digestion as previously described [27]. RINm5F cells were grown in RPMI 1640 tissue culture medium to a density of ∼75% prior to experimentation. The cells were then removed from growth flasks by treatment with 0.05% trypsin and 0.02% EDTA for 5 min at 37 °C, washed two times with complete CMRL-1066 media, and plated in CMRL-1066 at the indicated density.

Human islets were obtained from the Islet Cell Resource Center at Washington University School of Medicine (St. Louis, Missouri, United States). Before experiments, the human islets were cultured for 48 h at 37 °C in complete CMRL-1066 tissue culture medium in an atmosphere of 95% air and 5% CO_2_.

### Cell Death Assays

Cell viability was examined using the MTT assay and neutral red uptake. The MTT assay is based on the ability of viable cells to reduce MTT to insoluble formazan crystals [28]. For this assay, RINm5F cells or rat islet cells (1 × 10^5^ cells in 200 μl of complete CMRL-1066) were washed with tissue culture medium following treatments, and MTT was added at a final concentration of 0.5 mg/ml. Following an incubation for 3 h at 37 °C, the supernatants were removed and discarded, and the cells were lysed with acid-isopropanol (100 μl of 0.04 N HCl in isopropanol) and mixed thoroughly to dissolve the crystals for 5 min at room temperature. The optical density of this product was determined at a wavelength of 570 nm.

Cell viability was also evaluated by examining the accumulation of neutral red dye in viable cells [29]. For this assay, RINm5F cells or dispersed rat islet cells (1 × 10^5^ cells in 200 μl of complete CMRL-1066) were cultured for 24 or 48 h with the indicated concentrations of IL-1, camptothecin, or staurosporine; the culture supernatants were removed and discarded; and the cells were incubated in fresh medium containing 50 μg/ml neutral red at 37 °C. Following a 2-h incubation, the supernatant was removed, the cells were washed with a 1% formaldehyde-1% CaCl_2_ solution, and the neutral red dye was extracted in 100 μl of a 50% ethanol-1% acetic acid lysing solution. The accumulation of neutral red dye in the lysing solution was measured at a wavelength of 570 nm. Cell viability using both the MTT and neutral red assays is expressed as the percent of dead cells.

### Annexin V-FITC Staining

RINm5F cells or rat islet cells (1 × 10^5^ cells in 200 μl of complete CMRL-1066) were treated as indicated in the figure legends. Cells were removed from plates by cell scraping (RINm5F) or washing (islet cells). Externalization of phosphatidylserine (PS) was evaluated by annexin V staining according to manufacturer's instructions (Calbiochem) and quantified by flow cytometry [30].

### TUNEL Staining/Immunohistochemistry

RINm5F cells or rat islet cells (4 × 10^5^ cells in 400 μl of complete CMRL-1066) were treated as indicated in the figure legends. The cells were removed from plates and centrifuged onto glass slides. DNA damage was quantified by TUNEL staining according to the manufacturer's instructions using the In Situ Cell Death Detection Kit, Fluorescein (Roche). Cells containing DNA damage were colocalized with DAPI by incubating cells with DAPI (5 μg/ml) for 15 min followed by three washes with PBS (pH 7.4). DNA damage in rat islet cells was colocalized with insulin-containing cells as outlined previously [31].

### Caspase-3 Activity

RINm5F cells or rat islet cells (4 × 10^5^ cells in 400 μl of complete CMRL-1066) were treated as indicated in the figure legends. Cells were isolated and lysed, and capase-3 activity in the cell lysate was determined according to the manufacturer's instructions (Caspase-3 Fluorometric Assay Kit, R&D Systems). Caspase-3 activity was normalized to protein content and is presented as the fold-increase over the level of activity measured in the untreated control.

### Western Blot Analysis

Protein lysates prepared from RINm5F cells and rat islets were separated by SDS-PAGE and then transferred to nitrocellulose membranes (Amersham Pharmacia Biotech) under semidry transfer conditions as previously described [32]. Antigen was detected by enhanced chemiluminescence according to the manufacturer's specifications (Amersham Pharmacia Biotech). Antibody dilutions were: rabbit anti-mouse HMGB1, 1:1,000; and HRP-conjugated donkey anti-rabbit, 1:7,000.

### Nitrite Determination

Nitrite production was determined by mixing 50 μl of cell culture medium with 50 μl of Griess reagent [33]. The absorbance at 540 nm was measured, and nitrite concentrations were calculated from a sodium nitrite standard curve.

### Statistics

Statistical comparisons were made between groups using one-way ANOVA. Significant differences between groups (*p* < 0.05) were determined by Newman-Keuls post-hoc analysis.

## Results

### Nitric Oxide Mediates Cytokine-Induced Islet Cell Death

While it is clear that the inhibitory actions of cytokines such as IL-1 on glucose-stimulated insulin secretion are mediated by β-cell production of nitric oxide, the role of nitric oxide in islet cell death has been debated [8,18,19]. Short exposures (24–48 h) of islets and insulinoma cell lines to IL-1 alone, or in combination with IFN-γ, and TNF cause islet cell DNA damage and reduced oxidative capacity in a nitric oxide-dependent fashion [34–36]; however, prolonged exposures (7–9 d) of islets or purified β-cells with IL-1 or cytokine combinations have been reported to induce β-cell apoptosis in a nitric oxide-independent manner [19,22]. To investigate the mechanisms of cytokine-induced β-cell death, we initially examined whether IL-1 reduces β-cell viability using the MTT assay. Following a 24-h incubation with IL-1, death of RINm5F cells increased ∼20% (Figure 1A), and this cell death correlated with a ∼10-fold increase in production of nitrite, an oxidative metabolite of nitric oxide (Figure 1B). Following a 48-h incubation with IL-1, RINm5F cell death increased to ∼40% (Figure 1A), and cell death correlated with a ∼20-fold increase in nitrite production (Figure 1B). The NOS inhibitor NMMA prevented nitrite production (Figure 1B) and significantly attenuated IL-1-induced RINm5F cell death (Figure 1A). Similar to its effect in RINm5F cells, IL-1 induced the death of ∼25% of islet cells following 24- and 48-h incubations (Figure 1C). IL-1-induced islet cell death correlated with an increase in nitric oxide production (Figure 1D), and NMMA attenuated both islet cell death and nitric oxide production. These findings suggest that IL-1 induces islet and RINm5F cell death, that cell death correlates with the production of nitric oxide, and that cell death is attenuated by the inhibition of NOS.

**Figure 1:**
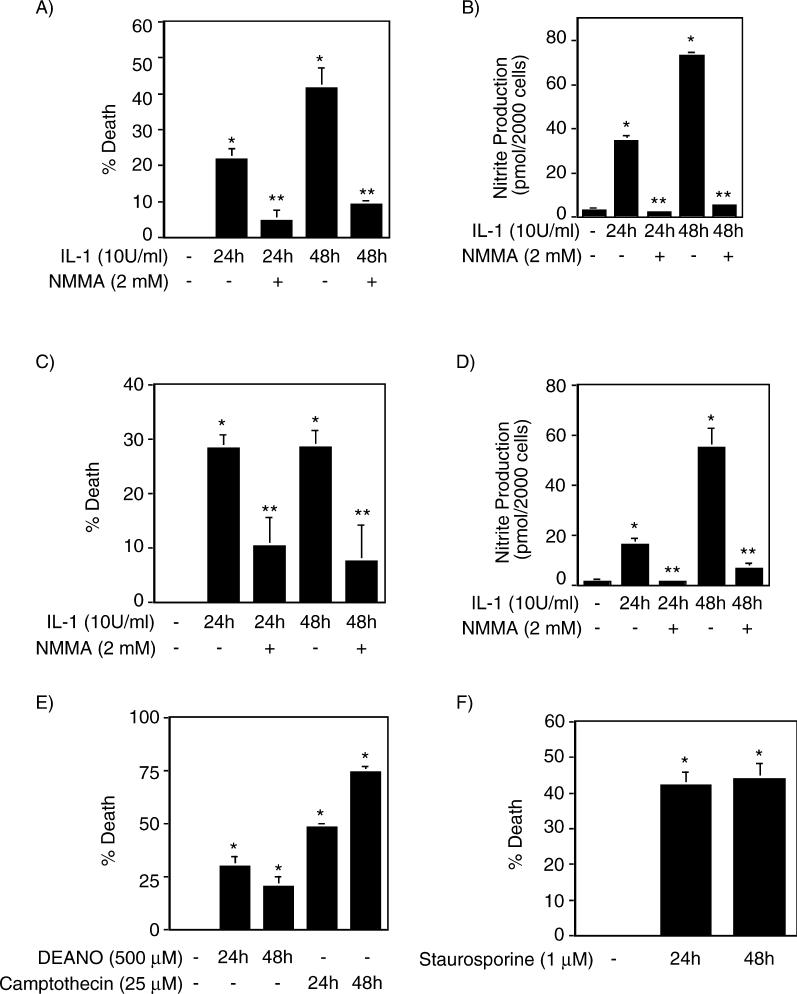
Nitric Oxide Mediates IL-1-Induced β-Cell Death as Determined by the MTT Assay RINm5F cells (A and B) or rat islets (C and D) were treated with IL-1 (10 units/ml) and NMMA (2 mM) for either 24 or 48 h, as indicated. The cells were isolated and viability determined using the MTT assay (A and C). The culture supernatants were isolated and nitrite production was determined using the Griess assay (B and D). (E and F) The effects of exogenously produced nitric oxide supplied by the donor compound DEANO (500 μM) and the apoptosis inducer camptothecin (25 μM) on RINm5F cell viability (E) and the apoptosis inducer staurosporine on rat islet cell viability (F) were determined by MTT assay. Cell viability data is expressed as percent death. Results for cell viability and nitrite production are the average ± standard error of the mean (SEM) of three independent experiments. *p < 0.05, significantly different from untreated controls. **p < 0.05, significantly different from IL-1-treated condition.

To examine whether nitric oxide can directly modulate cell viability, the effects of exogenously supplied nitric oxide on RINm5F cell viability were examined. RINm5F cells were chosen for these studies because they represent a homogenous population of β-cells, and the effects of cytokines on these cells have been characterized in detail [9]. Treatment of RINm5F cells for 24 h with the nitric oxide donor compound DEANO resulted in a 25% increase in cell death (Figure 1E). Following a 48-h exposure to DEANO, the level of death was not further increased, a finding that is likely due to the rapid decomposition of DEANO (which has a half-life of ∼2 min under these conditions). The effects of DEANO were compared to known inducers of apoptosis. The topoisomerase inhibitor camptothecin is a classical inducer of apoptosis in rapidly proliferating cell lines [37] and, as shown in Figure 1E, induces the death of ∼40% and 75% of RINm5F cells following 24- and 48-h incubations, respectively. Camptothecin is not an effective inducer of islet cell death (most likely due to the quiescent nature of these cells [unpublished data]); however, the kinase inhibitor staurosporine, another known inducer of apoptosis [38], stimulated the death of ∼40% of islet cells following a 24-h incubation (Figure 1F).

A second assay of cell viability, neutral red uptake, was used to confirm the observations that cytokines induce β-cell death (Figure 2). This assay is based on the ability of viable cells to accumulate neutral red dye, while nonviable cells fail to accumulate this dye [29]. Similar to the results presented for the MTT assay in Figure 1, IL-1 reduced RINm5F cell viability by ∼20%–25% following 24- or 48-h incubations, and cell death in response to this cytokine was attenuated by NMMA (Figure 2A). The apoptosis inducer camptothecin also reduced RINm5F cell viability by ∼60%–80% following 24- and 48-h incubations, as determined by neutral red uptake (Figure 2B). These findings provide evidence, in two different assays of cell death, that IL-1 reduces β-cell viability and that the loss of viability is mediated, in part, by the production of nitric oxide.

**Figure 2:**
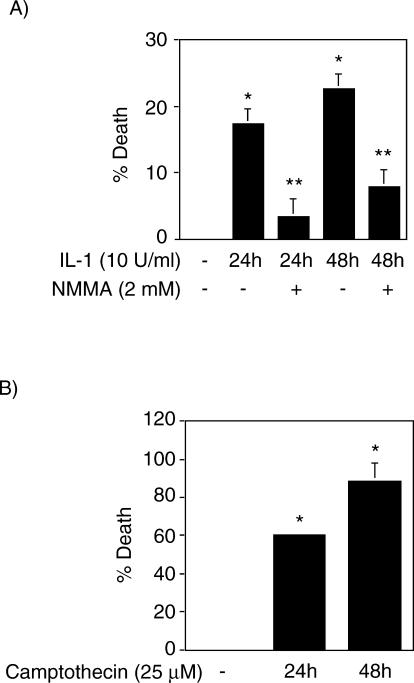
Effects of IL-1 on β-Cell Viability as Determined by Neutral Red Uptake RINm5F cells were treated for 24 h or 48 h with IL-1 (10 units/ml), NMMA (2 mM) (A), or camptothecin (25 μM) (B), and the cells were isolated and percentage of death determined using the neutral red uptake assay. Results are average ± SEM of three independent experiments. *p < 0.05, significantly different from untreated controls. **p < 0.05, significantly different from IL-1-treated condition.

### Morphological Analysis of IL-1-Induced β-Cell Death

While performing the cell viability experiments, clear morphological differences were observed for RINm5F cells treated with IL-1 as compared to the apoptosis inducer camptothecin. Control, untreated RINm5F cells grew as a flat monolayer of cells with distinct cell borders (Figure 3A). Treatment with IL-1 resulted in morphological changes that included cell swelling and the appearance of holes in the cytoplasm (Figure 3B), changes that are consistent with a necrotic form of cell death. Nitric oxide appears to be responsible for the morphological changes induced by IL-1, as NMMA attenuated RINm5F cell swelling and the formation of cytoplasmic holes (Figure 3C). The morphological changes in RINm5F cells undergoing apoptosis were strikingly different from the alterations induced by IL-1. RINm5F cells that underwent apoptosis following a 24-h treatment with camptothecin were severely shrunken and contained compacted nuclei, consistent with an apoptotic morphology (Figure 3D). This morphological analysis of the effects of IL-1 on RINm5F cell viability suggests that cytokine-induced β-cell death is not caused by classical apoptotic mechanisms and may occur by necrotic mechanisms.

**Figure 3:**
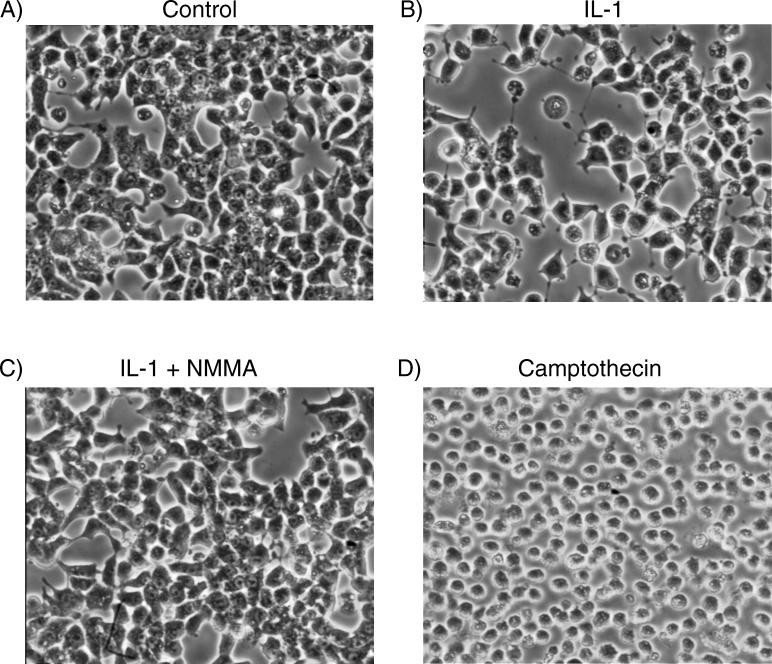
Morphological Analysis of IL-1-Induced RINm5F Cell Death RINm5F cells were treated for 24 h with IL-1 (10 units/ml) (B and C), NMMA (2 mM) (C), or camptothecin (25 μM) (D), and cellular morphology was examined by phase-contrast microscopy. Treatment with IL-1 resulted in cell swelling and detachment (B), events that are prevented by NMMA (C). In contrast to the effects of IL-1, induction of apoptosis using camptothecin (D) resulted in cell shrinkage and condensation of the nucleus. Untreated control cells are shown in (A). Results are representative of three independent experiments.

### IL-1 Stimulates β-cell DNA Damage in a Nitric Oxide-Dependent Manner

The TUNEL assay is commonly used to detect DNA damage in cells, and the results have frequently been used as evidence to support an apoptotic pathway of cell death. Using the TUNEL assay, the effects of IL-1 on the integrity of RINm5F and rat islet cell DNA were examined. In this assay, DNA strand breaks were detected by the incorporation of fluorescein dUTP (green fluorescence, Figure 4), and nuclei were visualized by co-staining with DAPI (blue fluorescence, Figure 4) or colocalized with insulin-containing islet cells (using a CY3-conjugated secondary antibody; red fluorescence, Figure 5). Treatment of RINm5F cells for 24 h with IL-1 resulted in DNA damage in ∼50% of the cells (see Figure 4B), and the level of DNA damage increased to ∼65% of the cells following a 48-h incubation (see Figure 4C). Importantly, IL-1-induced DNA damage was attenuated by NMMA to levels similar to those observed in untreated control cells (∼5%–8% TUNEL-positive; see Figure 4E and 4F). Representative fluorescent micrographs indicate that the morphology of TUNEL-positive RINm5F cells following treatment with IL-1 is significantly different from the morphology of cells treated with the apoptosis inducer camptothecin. While TUNEL staining in most RINm5F cells treated with IL-1 appeared uniform throughout the nuclei and colocalized with DAPI staining, there were a number of RINm5F cells in which the TUNEL staining appears to spill out of the nucleus (see Figure 4B and 4C, white arrows), indicating a loss of nuclear membrane integrity. Camptothecin stimulated DNA damage in ∼60% of the RINm5F cells, as shown in Figure 4D (white arrow), and the pattern of TUNEL and DAPI staining was distinctly different from that observed following IL-1 treatment. Camptothecin-induced TUNEL and DAPI staining appears more intense and punctate, indicating that chromatin condensation has occurred.

**Figure 4:**
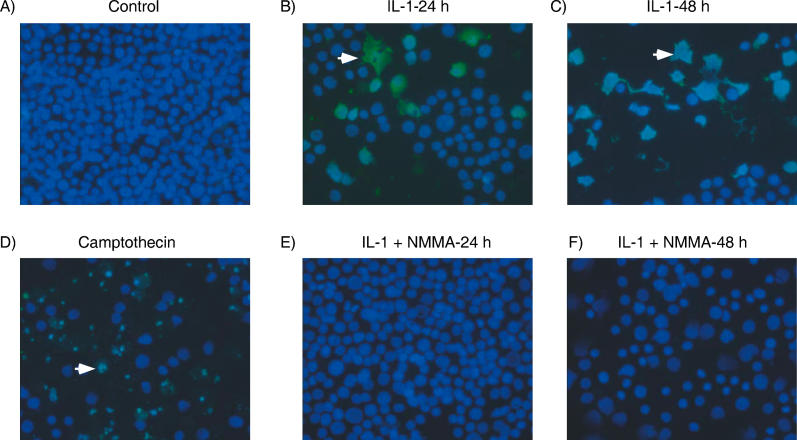
IL-1 Stimulates RINm5F Cell DNA Damage in a Nitric Oxide-Dependent Manner Treatment of RINm5F cells for 24 h (B) or 48 h (C) with IL-1 (10 units/ml) resulted in DNA damage, as determined by TUNEL staining (green fluorescence). DNA damage was attenuated by NMMA (E and F). The apoptosis inducer camptothecin (25 μM) stimulated DNA damage (D), and this DNA damage is morphologically distinct from that induced by IL-1. Nuclei are stained with DAPI (blue). The morphological differences in TUNEL staining, the loss of nuclear membrane integrity in response to IL-1 (B and C) and the condensation of DNA in response to camptothecin (D) are highlighted using white arrows. The levels of DNA damage in control cells (A) were less than 5%. Results are representative of three independent experiments.

**Figure 5:**
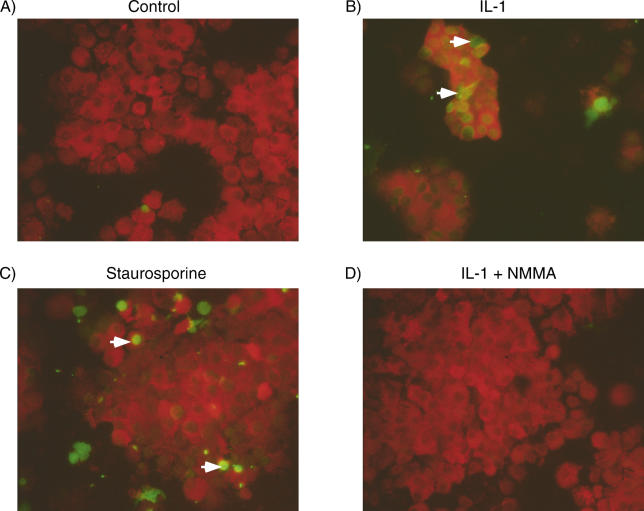
IL-1 Stimulates Islet Cell DNA Damage in a Nitric Oxide-Dependent Manner Treatment of rat islet cells for 24 h with IL-1 resulted in DNA damage as determined by TUNEL staining, and ∼95% of this damage occurred in insulin-containing cells (red fluorescence) (B). Nitric oxide appears to mediate IL-1-induced DNA damage, as it was attenuated by the NOS inhibitor NMMA (2 mM) (D). The apoptosis inducer staurosporine (1 μM) also stimulated DNA damage in both insulin containing and non-insulin containing cells (C). Less than 10% of control, untreated islet cells were TUNEL-positive (A). Results are representative of three independent experiments.

Similar to RINm5F cells, treatment of rat islets for 48 h with IL-1 resulted in DNA damage in ∼50% of islet cells, and over 95% of this DNA damage was found in β-cells, as determined by immunolocalization of TUNEL-positive cells with insulin-containing cells (Figure 5B). IL-1-induced DNA damage was prevented by NMMA (Figure 5D), indicating that nitric oxide is primarily responsible for the DNA damage observed in rat islet cells treated with IL-1. IL-1-induced DNA damage was compared with damage caused by the apoptosis inducer staurosporine. Treatment of rat islets for 48 h with staurosporine resulted in TUNEL-positive staining in ∼50% of islet cells, and this DNA damage was observed in both insulin-containing and non-insulin-containing cells (Figure 5C). Similar to RINm5F cells (see Figure 4), the morphology of TUNEL staining in the IL-1-treated islet cells appears uniform throughout the nucleus (Figure 5B, white arrows). In contrast, staurosporine-induced TUNEL staining in islet cells was more intense and punctate (Figure 5C, white arrows). Taken together, these findings suggest that IL-1 stimulates DNA damage in β-cells, and that nitric oxide is a primary mediator of this damage. In addition, these findings indicate that DNA damage induced by IL-1 results in morphological changes in β-cells that are markedly different from the DNA damage observed in β-cells undergoing staurosporine-induced apoptosis.

### IL-1 Fails to Activate Caspase-3 in RINm5F Cells or Rat Islets

To directly examine whether IL-1 induces classical apoptosis in rat islets or RINm5F cells, the effects of this cytokine on the activation of caspase-3 were examined. We decided to examine caspase-3 because it is an executioner caspase that is a downstream target of caspases involved in both the intrinsic and extrinsic pathways of apoptosis [39,40]. IL-1 failed to stimulate caspase-3 activation in RINm5F cells (Figure 6A) or rat islets (Figure 6B) treated for either 24 or 48 h. Since nitric oxide is a known inhibitor of caspase activity [41–43], the effects of IL-1 alone and in combination with NMMA on caspase-3 activity were examined. In the presence of NMMA, IL-1 still failed to stimulate caspase-3 activation in RINm5F cells or rat islets. In contrast to the lack of caspase-3 activation in response to IL-1, camptothecin induced a ∼9-fold increase in caspase-3 activity in RINm5F cells (Figure 6A), and staurosporine stimulated a ∼5-fold increase in caspase-3 activity in rat islets (Figure 6B).

**Figure 6:**
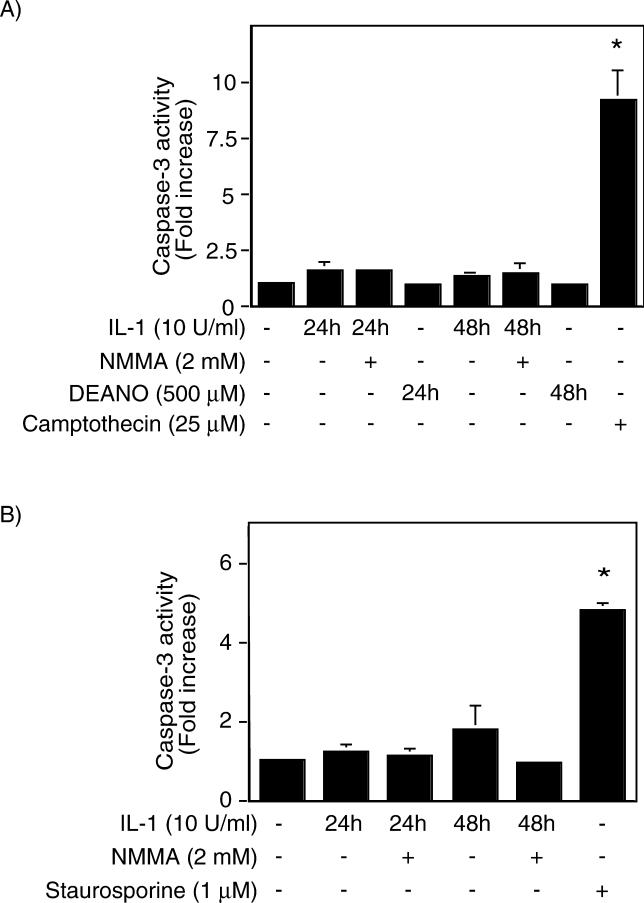
IL-1 Fails to Activate Caspase-3 in RINm5F Cells or Rat Islets (A) IL-1 (10 units/ml), alone or in combination with NMMA (2 mM) and the nitric oxide donor DEANO (500 μM), failed to activate caspase-3 in RINm5F cells following a 24- or 48-h exposure. As a positive control, the apoptosis inducer camptothecin (25 μM) stimulated a 9-fold increase in RINm5F cell caspase-3 activity. (B) Similar to RINm5F cells, in rat islets IL-1 (10 units/ml) alone or in combination with NMMA (2 mM) failed to activate caspase-3 following a 24- or 48-h incubation. The apoptosis inducer staurosporine (1 μM) stimulated a 5-fold increase in rat islet caspase-3 activity. Results are the average ± SEM of three independent experiments. *p < 0.05, significantly different from untreated control.

### Effects of IL-1 on Annexin V Staining in RINm5F Cells and Rat Islets

The externalization of PS on the outer membrane surface of cells is a hallmark of cells undergoing early or intermediate stages of apoptosis that can be quantified by flow cytometry using the calcium-dependent phospholipid-binding protein annexin V [30]. Using this assay, we show that IL-1 fails to stimulate PS externalization on RINm5F cells (Figure 7A) or rat islet cells (Figure 7B). In contrast, ∼55% of RINm5F cells stained positive for annexin V following a 24 or 48-h incubation with camptothecin, and ∼15% of rat islet cells stained positive for annexin V following a 24- or 48-h incubation with staurosporine. The absence of annexin V staining on rat islet cells and RINm5F cells treated with IL-1 is consistent with the lack of caspase-3 activation, and suggests that, while IL-1 stimulates β-cell death, it does not appear to occur by classical apoptotic mechanisms.

**Figure 7:**
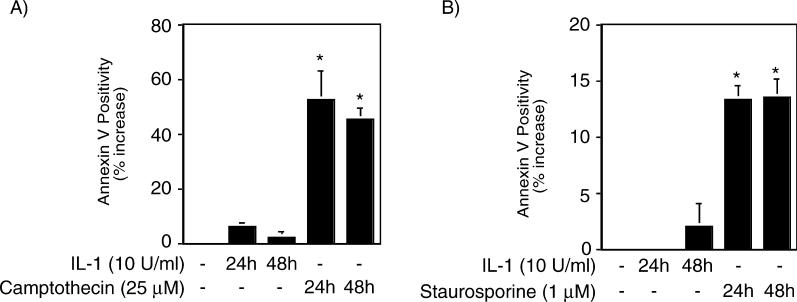
Effects of IL-1 on Annexin V Staining in RINm5F Cells and Rat Islets Treatment of RINm5F cells (A) or rat islet cells (B) with 10 units/ml IL-1 did not stimulate PS externalization, as determined by annexin V staining and quantified by flow cytometry. In contrast, treatment of RINm5F Cells with camptothecin (25 μM) (A) or rat islet cells with staurosporine (1 μM) (B) stimulated the externalization of PS. Results are presented as percent increase in annexin V-positive cells above the basal levels (∼3%–7%) detected on untreated control RINm5F or rat islet cells and are the average ± SEM of three independent experiments. *p < 0.05, significantly different from untreated control.

### Biochemical Evidence that IL-1 Stimulates β-Cell Necrosis

HMGB1 is a chromatin-binding protein that is selectively released by cells undergoing necrosis [26,44–46]. It is not released from cells undergoing apoptosis, as it is sequestered by chromatin [45]. To determine whether IL-1 stimulates β-cell necrosis, RINm5F cells were treated for 24 or 48 h in the presence or absence of NMMA, and the supernatants were isolated and examined by Western blot analysis for the presence of HMGB1. As shown in Figure 8A, IL-1 stimulates the release of low levels of HMGB1 by RINm5F cells, which is first apparent following a 24-h incubation, with higher levels released following a 48-h incubation. In a similar manner, IL-1 stimulated the release of HMGB1 by rat islets (Figure 8B). NMMA attenuates IL-1-induced HMGB1 release by both rat islets and RINm5F cells, indicating that nitric oxide is a mediator of β-cell necrosis. To confirm that nitric oxide induces β-cell necrosis, we show that a 24-h incubation of RINm5F cells (Figure 8C) or rat islets (Figure 8D) with the nitric oxide donor DEANO results in HMGB1 release. In contrast, barely detectable levels of HMGB1 were released by RINm5F cells treated with camptothecin or islets incubated with staurosporine for 24 h (Figures 8C and 8D). As a control, inactivated DEANO failed to stimulate HMGB1 release by either rat islets or RINm5F cells (unpublished data). The release of HMGB1 by rat islets and RINm5F cells provides biochemical evidence that IL-1 stimulates β-cell death by necrotic mechanisms.

**Figure 8:**
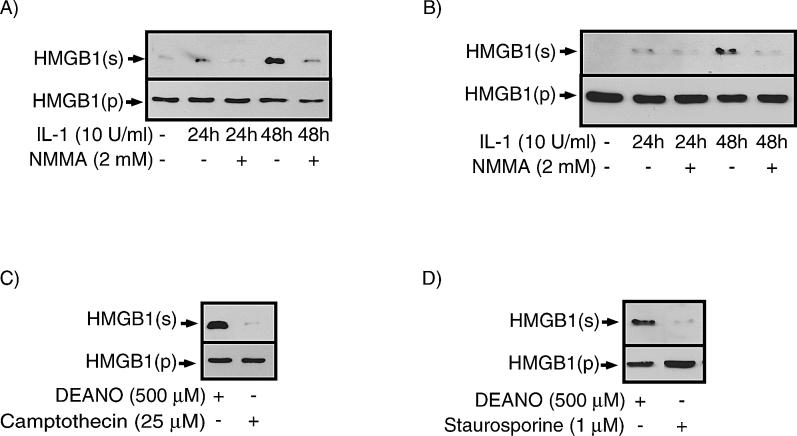
IL-1 Stimulates HMGB1 Release in RINm5F Cells and Rat Islets (A and B) RINm5F cells (A) or rat islets (B) were treated for 24 or 48 h with 10 units/ml of IL-1 and 2 mM NMMA as indicated. The levels of HMGB1 released into the supernatant (s) and contained in the cells (p) were determined by Western blot analysis. Results show that IL-1 stimulates the release of HMGB1 from RINm5F cells and rat islets, and that this release is attenuated by the NOS inhibitor NMMA. (C) Treatment of RINm5F cells for 24 h with DEANO (500 μM) stimulated HMGB1 release, while the apoptosis inducer camptothecin (25 μM) failed to stimulate HMGB1 release from RINm5F cells. (D) Nitric oxide supplied exogenously via treatment with DEANO (500 μM) also stimulated HMGB1 release by rat islets, while HMGB1 was not released under conditions of β-cell apoptosis in response to treatment with staurosporine. Results are representative of three independent experiments.

### Effect of Caspase-3 Inhibition on Cytokine-Induced β-Cell Death

To further explore the mechanisms of β-cell death, the effects of caspase-3 inhibition on β-cell death in response to apoptosis inducers and IL-1 were examined. As expected, the caspase-3 inhibitor Z-DQMD-CHO attenuated camptothecin-induced RINm5F cell death, as determined by MTT assay (Figure 9A), annexin V staining (Figure 9B), and caspase-3 activation (unpublished data). In contrast, inhibition of caspase-3 failed to attenuate IL-1-induced RINm5F cell death following a 24- or 48-h incubation (Figure 9C). Furthermore, caspase-3 inhibition failed to prevent RINm5F cell HMGB1 release induced by the exogenous addition of nitric oxide by the donor DEANO (Figure 9D), or following a 48-h incubation with IL-1 (Figure 9E). These findings demonstrate that β-cell death in response to IL-1 treatment does not require the activation of caspase-3, an executioner caspase, and that caspase-3 inhibition does not prevent β-cell necrosis in response to IL-1 treatment or exposure to exogenous nitric oxide, as assessed by the release of HMGB1.

**Figure 9:**
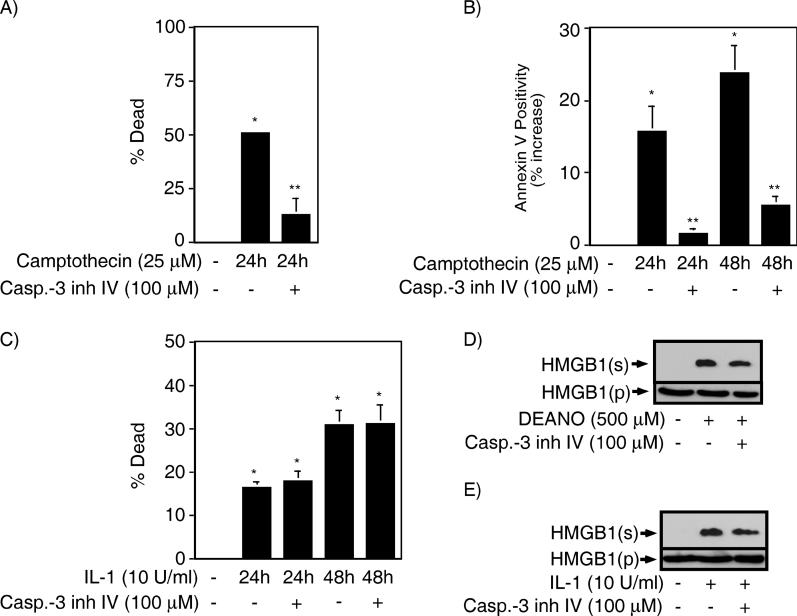
Caspase-3 Inhibition Prevents β-Cell Apoptosis but Does Not Prevent IL-1-Induced Necrosis The caspase-3 inhibitor Z-DQMD-CHO (100 μM) attenuated RINm5F cell death induced by a 24-h incubation with camptothecin as determined by MTT assay (A) or annexin V staining (B). This caspase-3 inhibitor failed to prevent RINm5F cell death in response to a 48-h incubation with 10 units/ml IL-1 as determined by the MTT assay (C). Z-DQMD-CHO also did not attenuate HMGB1 release by RINm5F cells treated for 24 h with 500 μM DEANO (D) or for 48 h with 10 units/ml IL-1 (E). The levels of HMGB1 released into the supernatant (s) and contained in the cells (p) were determined by Western blot analysis. Results for MTT assay (A and B) and annexin V staining (C) are the average ± SEM of three independent experiments. *p < 0.05, significantly different from untreated control. **p < 0.05, significantly different from camptothecin-treated condition. Results for HMGB1 release are representative of three independent experiments.

### Cytokines Stimulate HMGB1 Release from Human Islets in a Nitric Oxide-Dependent Manner

In contrast to the effects of IL-1 on rat islets, a combination of cytokines (IL-1, TNF, and IFN-γ) is required to stimulate nitric oxide synthase expression and nitric oxide production from human islets. Previous studies have shown that, individually, these cytokines fail to stimulate iNOS expression or nitric oxide production, or to induce human islet cell death [47–49]. To examine whether nitric oxide modulates human islet viability, we examined the effects of a 48-h incubation of human islets with IL-1, IFN-γ, and TNF on the release of HMGB1. Treatment of human islets with this cytokine mixture stimulated a ∼7 fold increase in nitrite production (Figure 10A), and nitric oxide production correlated with high levels of iNOS expression (unpublished observation). Importantly, under conditions in which human islets produce nitric oxide, HMGB1 accumulated in the supernatant of these islet cultures (Figure 10B). HMGB1 release appears to be dependent on the production of nitric oxide, as the iNOS selective inhibitor AG attenuated the production of nitric oxide and the release of HMGB1 by human islets. The apoptosis inducer staurosporine failed to stimulate the production of nitric oxide or release of HMGB1. Alone, neither IL-1, TNF, nor IFN-γ stimulated nitric oxide production, iNOS expression, or the release of HMGB1 from human islets following a 24- or 48-h incubation (unpublished data), providing evidence that the increased production of nitric oxide by human islets in response to the combination of cytokines is required for the release of HMGB1 by human islets. Overall, these findings are consistent with the effects of IL-1 on rat islets and RINm5F cells, in that they provide direct evidence that nitric oxide mediates the release of HMGB1, a marker of necrotic cell death, by human islets in response to cytokine treatment.

**Figure 10:**
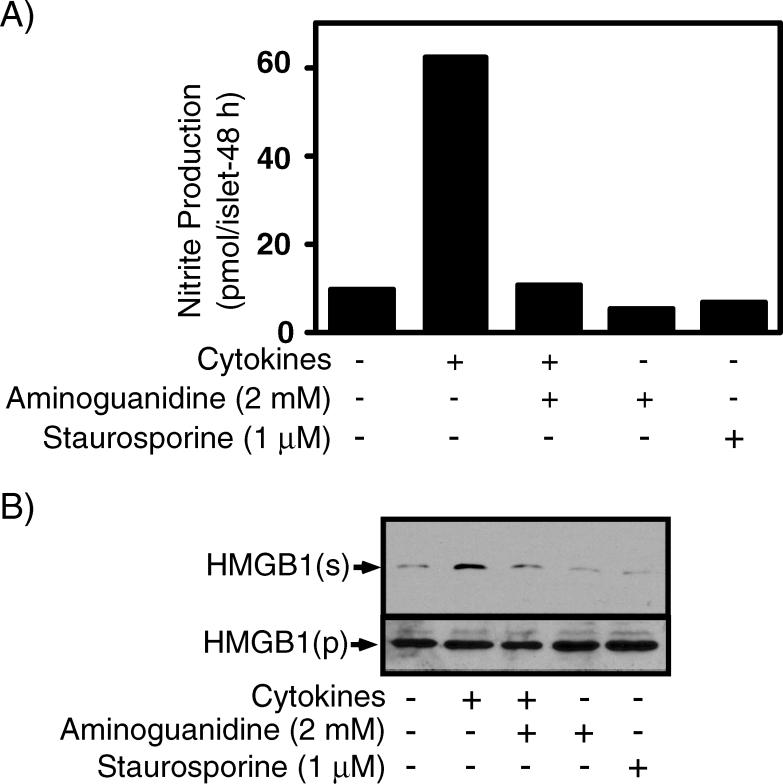
Nitric Oxide-Dependent Release of HMGB1 from Cytokine-Treated Human Islets Human islets (200 islets in 400 μl of complete CMRL-1066) were treated with a combination of IL-1 (50 units/ml), IFN-γ (500 units/ml), and TNF (50 nM) in the presence or absence of the NOS inhibitor AG (2 mM) or treated with staurosporine (1 μM) alone. (A) Following 48h -incubation the supernatant was removed and nitrite production was determined using the Griess assay. The levels of HMGB1 released into the supernatant (s) and contained in the islets (p) were determined by Western blot analysis. (B) Results indicate that cytokine-stimulated HMGB1 release correlates with nitric oxide production, and that inhibition of nitric oxide production using AG attenuates HMGB1 release from human islets. Similar results have been obtained in three of five independent preparations of human islets.

## Discussion

Autoimmune diabetes is characterized by an inability to regulate blood glucose levels due to the autoimmune destruction of insulin-producing pancreatic β-cells. The development of autoimmune diabetes is a T cell-dependent process, as they represent a major cellular constituent found in insulitis, and it is possible to prevent diabetes by T cell depletion [2,3,50]. While T cells are responsible for killing the majority of β-cells during the development of diabetes, relatively little is known concerning the events that initiate this autoimmune response directed against β-cells. Mathis and coworkers [3,51] have recently proposed that β-cell death during normal physiological islet remodeling, which peaks at 14–17 d after birth, may supply β-cell antigens that can then be engulfed by dendritic cells. These antigen-presenting cells (APCs) then ferry the antigen to pancreatic lymph nodes, where they may activate naïve diabetogenic T cells [3,51]. Although the mechanisms of β-cell death during islet remodeling are likely apoptotic, it is possible to mount a T cell response by antigens produced by cells undergoing apoptotic cell death [52].

While experimental evidence supports this model for initiation of diabetes in the NOD mouse [51], this model does not take into account the low concordance rate and variable time of onset of diabetes observed in identical twins [53,54]. Specifically, if β-cell death during normal physiological islet remodeling supplies antigens that activate naïve diabetogenic T cells, one would have to assume that the extent/efficiency of β-cell death and/or antigen processing and presentation to naïve T cells may be highly variable in individuals with an identical genetic makeup. An alternative hypothesis to explain the initiation of autoimmune diabetes, proposed by Lacy [55,56], suggests that intraislet macrophage activation and local release of macrophage-derived inflammatory mediators initiates β-cell death, antigen release, and autoimmunity directed against β-cells. The initiating event leading to intraislet macrophage activation may be viral, bacterial, or chemical in origin. It is well known that viral or bacterial infection stimulates macrophage production of IL-1 and TNF, and these cytokines have been shown to impair β-cell function and induce β-cell death [4,5,57]. In support of this model, previous studies have shown that feeding rodents a diet deficient in essential free fatty acids, known to deplete resident macrophages [58,59], attenuates diabetes induced by the chemical toxin streptozotocin [60] and the natural progression of diabetes in BB rats [61]. In addition, a number of studies have shown that the local release of IL-1 by activated intraislet macrophages in rodent and human islets leads to β-cell dysfunction and damage [13,14,62].

In this report we have evaluated the cellular mechanisms by which the macrophage-derived cytokine IL-1 kills β-cells. Using three different methods to evaluate cell viability, MTT oxidation, neutral red uptake, and TUNEL staining (DNA damage), we show that IL-1 induces β-cell death (see Figures 1–3). Nitric oxide appears to be one mediator of β-cell death, as NOS inhibition attenuated cell death in response to IL-1, and nitric oxide, supplied exogenously using donor compounds, reduced β-cell viability. The mechanism by which IL-1 stimulates β-cell death is both morphologically and biochemically different from known inducers of apoptosis. Morphologically, IL-1-induced β-cell death is characterized by cell swelling and diffuse TUNEL staining–changes indicative of the loss of membrane integrity, or necrosis. In response to inducers of apoptosis, the morphology of dying β-cells differs drastically from the effects of IL-1. Apoptosis induction results in cell shrinkage and tight, punctate TUNEL staining, consistent with the form of cell death that we have observed with known inducers of apoptosis. To investigate biochemical mechanisms responsible for β-cell death, we evaluated the effects of IL-1 on caspase-3 activation and PS externalization (annexin staining). Caspase-3 activity was chosen because it the executioner caspase activated by initiator caspases involved in both extrinsic and intrinsic apoptotic pathways [39,40]. While many reports have suggested that IL-1 induces β-cell death by apoptosis, we show that IL-1 fails to activate caspase-3 or to stimulate annexin V staining in islets or RINm5F cells. In contrast, known apoptosis inducers stimulated 6- to 9-fold increases in caspase-3 activity and significantly increased annexin V staining on RINm5F cells and rat islet cells. The lack of RINm5F cell and islet caspase activity following treatment with IL-1 is not surprising, as IL-1 stimulates β-cell production of nitric oxide, and nitric oxide has been shown to inhibit caspase activity by S-nitrosation of the active site cysteine [41,43].

Since these findings suggest that the macrophage-derived cytokine IL-1 does not induce apoptosis, we sought to identify whether IL-1 kills β-cells by necrosis. The morphological changes (see Figure 3) and the diffuse TUNEL staining in RINm5F cells and rat islet cells (see Figures 4 and 5) induced by IL-1 treatment are consistent with a necrotic type of cell death. To provide biochemical evidence in support of IL-1-induced β-cell necrosis, the effects of this cytokine on the release of HMGB1 from β-cells was examined. HMGB1 is a chromatin-binding protein that functions to distort the double helix, allowing proper physical interactions between transcription factors and chromatin [63]. Recent studies have shown that HMGB1 is released from cells dying by necrosis, but it remains tightly bound to chromatin in cells undergoing apoptosis [26,44–46]. In this study, we show that IL-1 stimulates HMGB1 release from RINm5F cells and rat islets in a nitric oxide-dependent manner, and that HMGB1 release is not affected by the inhibition of caspase activity. These findings suggest that IL-1-induced HMGB1 release is mediated, at least in part, by nitric oxide, a conclusion further supported by the ability of nitric oxide to directly stimulate HMGB1 release by RINm5F cells and rat islets. Importantly, under conditions in which RINm5F cells or islets have been forced to undergo apoptosis, HMGB1 was not released. Overall, this morphological and biochemical evidence supports β-cell necrosis as the primary mechanism by which IL-1 induces β-cell death, and that this death pathway is mediated, in part, by nitric oxide.

While IL-1 is sufficient to inhibit the function and to induce the death of rat β-cells, alone, the exogenous addition of IL-1 does not inhibit the function or induce death of human β-cells [47,48]. A combination of the cytokines IL-1, TNF, and IFN-γ is required to impair human β-cell function and induce islet cell death [47,48]. Treatment of human islets for 24- or 48-h with IL-1 + IFN-γ + TNF results in inhibition of glucose-stimulated insulin secretion and mitochondrial aconitase activity, effects attenuated by the iNOS inhibitors NMMA and AG [47,64]. In addition, the local release of IL-1 by intraislet macrophages in response to TNF + IFN-γ + LPS treatment results in inhibition of glucose-stimulated insulin secretion that is attenuated by inhibitors of iNOS [14]. These findings support a role for nitric oxide as a mediator of cytokine-induced β-cell damage in human following exposures of 24 and 48 h. In contrast, extended incubation (for 6–9 d) of human islets with IL-1 + TNF + IFN-γ has been reported to inhibit insulin secretion and induce β-cell apoptosis in a nitric oxide-independent manner [19,48]. The latter findings have led to speculation that human islets are less sensitive to nitric oxide than are rodent islets [65]. Comparisons of cellular responses to cytokines across species are complicated by the genetic background of each species. This is especially true when using human islets in which the genetic background is different for each individual human islet preparation. In contrast, studies with rodent islets are usually performed with a specific strain of rat or mouse. Importantly, Nerup and coworkers have shown that the ability of IL-1 to inhibit β-cell function and to induce iNOS expression varies in three different rat strains [66]. These findings suggest that the genetic background, even within a given species of animal, contributes to the islet response to cytokines. Taking this into account, we have evaluated the effects of IL-1, TNF, and IFN-γ on nitric oxide production and HMGB1 release by human islets. In three of five preparations, the combination of cytokines stimulated HMGB1 release by human islets by a mechanism that was sensitive to inhibition by the iNOS inhibitors AG (Figure 10) and NMMA (unpublished data). Importantly, in two human islet preparations the combination of cytokines failed to stimulate HMGB1 release and also failed to stimulate nitric oxide production or iNOS expression. The reason for this lack of response to cytokines in two of the human islet preparations is unknown; however, it has been reported that elevated levels of heat-shock protein 70 attenuate the ability of β-cells to express iNOS and produce nitric oxide, and the strain differences in response of rat islets to IL-1 have been reported to be associated with differences in levels of heat-shock protein 70 expression [67–69]. Taking this into account, our findings suggest that when human islets produce sufficient levels of nitric oxide in response to cytokines, one consequence is the release of HMGB1. In contrast, when human islets fail to respond to cytokines by producing nitric oxide, they also do not release HMGB1.

HMGB1 is not only a marker of cells undergoing necrosis, but it is also immunologically active. It has been shown to bind to Toll2 and Toll4 receptors and to activate nuclear factor κB-dependent gene expression [70–72], and antagonists of HMGB1 have been shown to attenuate sepsis in animal models [73]. It is well established that necrotic cells release immunological adjuvants that activate APCs and that amplify and sustain T cell-dependent immune responses [74–76]. Recently, Rovere-Querini et al. showed that necrotic HMGB1-deficient cells have a reduced capacity to activate APCs, and that HMGB1 neutralization attenuates the ability of supernatants derived from necrotic wild-type cells to activate APCs [77]. HMGB1 also appears to enhance primary antibody responses to soluble antigens and to enhance cytotoxic lymphocyte responses [77]. While caution should be exercised when extrapolating in vitro findings to mechanisms of disease pathogenesis, it is tempting to speculate on the potential role of intraislet macrophage activation as a precipitating event in the development of autoimmune diabetes. In previous studies, we have shown that the 10–15 resident macrophages found in islets, when activated, produce IL-1 to levels sufficient to inhibit β-cell function in a manner similar to that occurring with the exogenous addition of this cytokine to islets [13,14,78]. We now show that, at these same concentrations, IL-1 kills β-cells by necrotic mechanisms that are associated with the release of the immunological adjuvant HMGB1–a molecule known to augment APC activation and cytolytic responses of T cells.

## Abbreviations

AG: aminoguanidine
APC: antigen-presenting cell
DAPI: 4′,6-diamidino-2-phenylindole dihydrochloride
DEANO: sodium (Z)-1(*N,N*-diethylamino) diazen-1-ium-1,2-diolate
HMGB1: high-mobility group box 1 protein
IFN: interferon
IL: interleukin
iNOS: inducible nitric oxide synthase
MTT: 3-(4,5-dimethylthiazol-2-yl)-2,5-diphenyl tetrazolium bromide
NMMA: N^G^-monomethyl-L-arginine
PS: phosphatidylserine
SEM: standard error of the mean
TNF: tumor necrosis factor
TUNEL: terminal nick-end labeling
